# Investigation on environmental factors contributing to bispecific antibody stability and the reversal of self-associated aggregates

**DOI:** 10.1186/s40643-024-00796-y

**Published:** 2024-08-23

**Authors:** Nattha Ingavat, Nuruljannah Dzulkiflie, Jia Min Liew, Xinhui Wang, Eunice Leong, Han Ping Loh, Say Kong Ng, Yuansheng Yang, Wei Zhang

**Affiliations:** 1 Downstream Processing Group, Bioprocessing Technology Institute, Agency for Science, Technology and Research (A*STAR), Singapore, Singapore; 2 Animal Cell Bioprocessing Group, Bioprocessing Technology Institute, Agency for Science, Technology and Research (A*STAR), Singapore, Singapore; 3 Cell Line Development Group, Bioprocessing Technology Institute, Agency for Science, Technology and Research (A*STAR), Singapore, Singapore

**Keywords:** Bispecific antibody, Fab-scFv, Stability, Reversible-self association, Environmental factors

## Abstract

Bispecific antibodies (bsAbs) hold promises for enhanced therapeutic potential surpassing that of their parental monoclonal antibodies. However, bsAbs pose great challenges in their manufacturing, and one of the common reasons is their susceptibility to aggregation. Building on previous studies demonstrating the functionality and potential manufacturability of Fab-scFv format bsAb, this investigation delved into the impact of environmental factors—such as pH, buffer types, ionic strength, protein concentrations, and temperatures—on its stability and the reversal of its self-associated aggregates. Mildly acidic, low-salt conditions were found optimal, ensuring bsAb stability for 30 days even at elevated temperature of 40 °C. Furthermore, these conditions facilitated the reversal of its self-associated aggregates to monomers during the initial 7-day incubation period. Our findings underscore the robustness and resilience of Fab-scFv format bsAb, further confirming its potential manufacturability despite its current absence as commercial products.

**Graphical Abstract:**

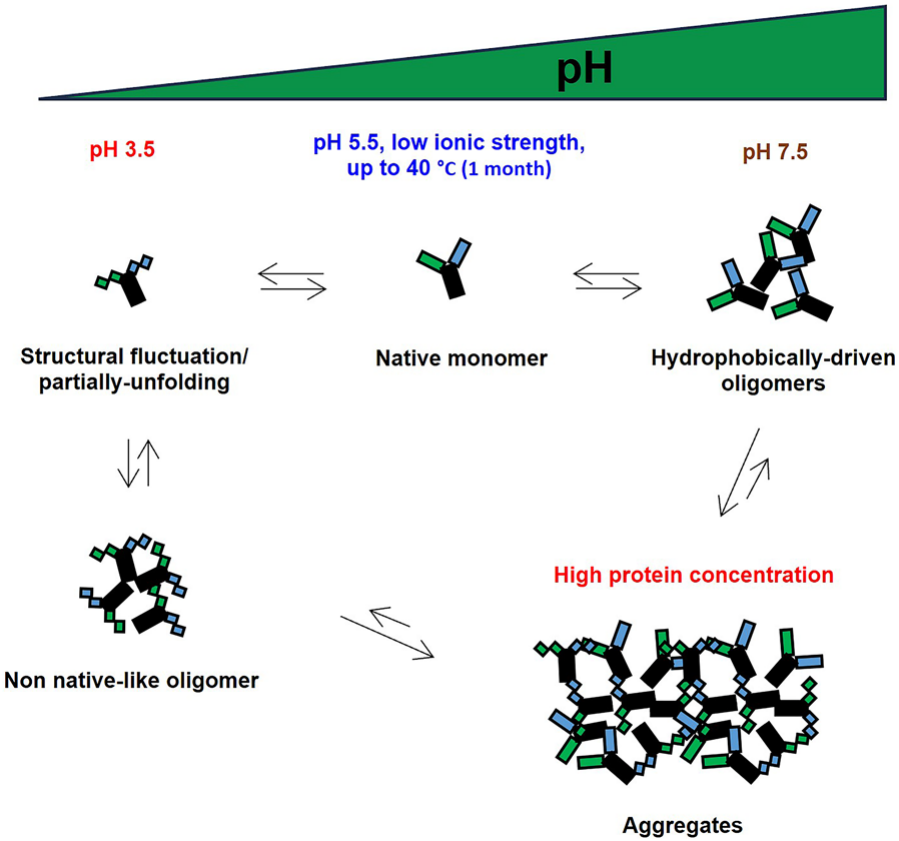

**Supplementary Information:**

The online version contains supplementary material available at 10.1186/s40643-024-00796-y.

## Introduction

Bispecific antibodies (bsAbs) represent a cutting-edge advancement over traditional monoclonal antibodies, intentionally designed to incorporate two distinct antigen-binding sites within a single molecule. This strategic engineering grants bsAbs significant enhancements in various aspects, including binding avidity to targets, overcoming drug resistance, and the ability to redirect cytotoxic effector cells (Sun et al. 2023; Labrijn et al. 2019). Consequently, bsAbs demonstrate remarkable therapeutic efficacy compared to their parental monoclonal counterparts. Moreover, bsAbs offer unique therapeutic potential beyond any combination of parental monoclonal antibodies (Labrijn et al. 2019; Chen and Zhang 2021; Rouet and Christ 2014; Tapia-Galisteo et al. 2023). As of now, 14 bsAbs have received approval for cancer treatment, with three additional bsAb drugs, chosen for non-oncology applications (Klein 2024).

BsAbs are classified into three primary formats (Labrijn et al. 2019; Chen and Zhang 2021). The fragment-based format, exemplified by bispecific T-cell engager (BiTE), represents a minimalist design devoid of the Fc region, featuring only antigen-binding domains and linkers (Labrijn et al. 2019). Notably, Blinatumomab, based on the BiTE format, achieved the distinction of being the first bsAb approved by the US FDA in 2014 (Wei et al. 2022) and by the EU in 2015 (Labrijn et al. 2019). Symmetric and asymmetric formats, resembling IgG-like bsAbs, are the other two categories (Chen and Zhang 2021). Symmetric bsAbs maintain a Fc region while evading chain association issues, typically designed as tetravalent structures (2 + 2). The same as symmetric bsAbs, asymmetric bsAbs also aim to closely mimic the native architecture of antibodies to preserve their functional characteristics and desirable quality attributes (Labrijn et al. 2019).

To date, asymmetric bsAbs under development surpass symmetric variants in number. They are typically composed of up to four polypeptide chains, including heavy chains (HCs) and light chains (LCs), derived from two distinct parental monoclonal antibodies. However, introducing structural asymmetry poses challenges with chain association and elevated impurity profiles. To address these challenges, technologies such as knob-into-hole (Ridgway et al. 1996) and electrostatic complementarity (Gunasekaran et al. 2010; Nardis et al. 2017) have been used to mitigate HC homodimerization. CrossMab (swapping HC and LC within a Fab domain) is one strategy commonly employed to facilitate heavy and light chain associations (Sun et al. 2023; Wei et al. 2022; Surowka and Klein 2024), and another popular strategy is to replace one antigen-binding fragment (Fab) domain with a single-chain variable fragment (scFv) domain (Chen and Zhang 2021) to form a Fab-scFv format bsAb.

Among diverse asymmetric bsAb formats currently under development, the Fab-scFv configuration stands out prominently (Panina et al. 2020; Bhatta et al. 2021). Numerous studies not only confirm its functionality but also underscore its potential manufacturability (Loh et al. 2023; Moretti et al. 2013; Suurs et al. 2019). Given this promising prospect, there is a need to investigate further how various environmental conditions affect its long-term stability, which could benefit Fab-scFv format bsAb process development and future manufacturing.

**Fig. 1:**
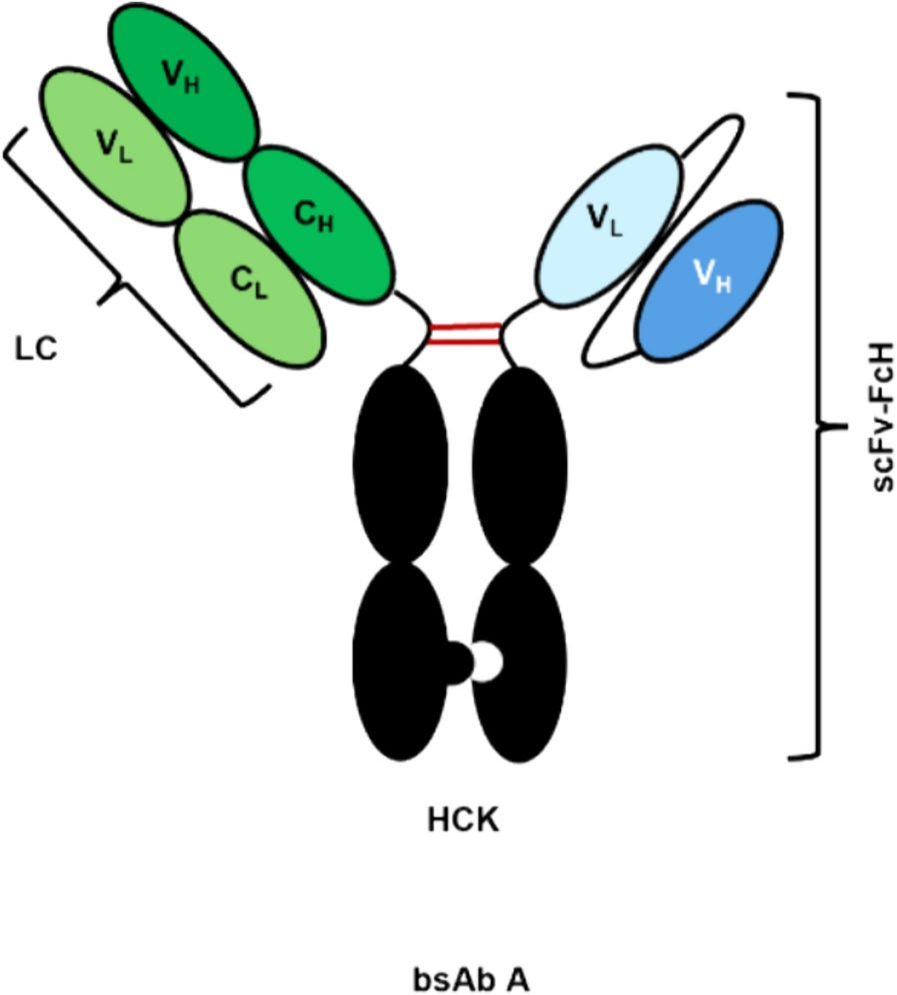
A model molecule, bsAb A, used in this study. BsAb A represents a Fab-scFv format asymmetric molecule with heterodimeric knob-in-to-hole Fc, featuring a Her2 binding moiety in its Fab domain (green) and the anti-CD3 moiety incorporated in its scFv domain (blue). Trastuzumab light chain (LC), trastuzumab heavy chain knob (HCK), and anti-CD3 scFv-Fc hole (scFv-FcH)

Utilizing a model Fab-scFv format bsAb, referred to as bsAb A (Fig. 1), this study investigated how extrinsic factors, including pH, types of buffer, ionic strength, protein concentration and temperature, influence bsAb stability and the reversal of self-associated aggregates. BsAb A features a Her2 binding moiety in its Fab domain and an anti-CD3 moiety incorporated in its scFv domain, utilizing knob-into-hole technology to mitigate HC mispairing issue. Previous research has highlighted Fab-scFv format bsAb A as one of the most promising bsAb molecules in terms of functionality and manufacturability compared to other tested bsAb formats (Loh et al. 2023). Understanding how extrinsic factors influence bsAb stability will help in designing suitable strategies during drug manufacturing, formulation, and storage to ensure bsAb stability from production to administration.

## Material and methods

### Materials

All buffers, salts, and reagents were purchased from Sigma-Aldrich except for disodium hydrogen phosphate, citric acid, histidine hydrochloride, and MES that were purchased from Merck Millipore. MabSelect™ PrismA was purchased from Cytiva.

### BsAb culture production

Stably transfected CHO K1 cell lines expressing Fab-scFv format bsAb molecules were generated through the site-specific integration of plasmid vectors, which carried genes encoding the trastuzumab light chain (LC), trastuzumab heavy chain knob (HCK), and anti-CD3 scFv-Fc hole (scFv-FcH) (Fig. 1). The cDNAs for trastuzumab variable fragment heavy chain (VH) and variable fragment light chain (VL), as well as anti-CD3 VL and VH, were designed using the amino acid sequences of trastuzumab and pasotuxizumab found in the international ImMunoGeneTics information system (IMGT). To facilitate heterodimeric Fc pairing, the CH3 domains in HCK and scFv-FcH were engineered to form a knob (through mutations of S354C:T366W) and a hole (through mutations of Y349C:T366S:L368A:Y407V), respectively, based on a previous study (Merchant et al. 1998). The VH and VL in scFv were connected through a flexible linker (G4S)3, which was further linked to FcH through a G4S linker.

Stably transfected pools were created through recombinase-mediated cassette exchange (RMCE), achieved by co-transfecting the CHO K1 master cells with a suitable targeting vector expressing a bsAb and a vector expressing FLPe. A detailed protocol for generating stably transfected pools and conducting production in fed-batch cultures can be found in our previous study (Ong et al. 2022). In brief, the cell lines which were stably transfected, were cultivated in EX-CELL® Advanced CHO Fed-batch Medium (SAFC, Sigma) and supplemented with 6 mM glutamine (Sigma) in 50 mL tubespin (TPP), placed in a humidified Kuhner shaker (Adolf Kühner AG) with 8% CO_2_ at 37 °C.

BsAb A was produced in a 2 L glass stirred tank (Biostat® B-DCU, Sartorius), using a 14-day fed-batch mode with temperature shift. The vessel was inoculated with 3.25 × 10^5^ cells/mL, with set points for temperature at 37 °C, pH 7.00 and 50% dissolved oxygen. On day 7, the temperature was reduced to and maintained at 34 °C until the end of culture. The pH was controlled using carbon dioxide gas directed to the overlay or 1 M sodium bicarbonate (Merck) solution. The dissolved oxygen concentration was controlled using air and oxygen directed to the microsparger. Basal medium used was EX-CELL Advanced CHO Fed-batch Medium (Merck). On days 3, 5, 7, 9, 11, EX-CELL Advanced CHO Feed 1, without glucose, (Merck) was fed to the vessel at a concentration of 10% (v/v). Glucose (Merck) was supplemented separately to 3 g/L using a concentrated stock of 400 g/L when the glucose concentration was below 2.5 g/L at the time of sampling. Samples were collected daily for viable cell density (Vi-CELL XR, Beckman Coulter), nutrient and metabolite (BioProfile 100 plus, Nova Biomedical), osmolality (Vapor Pressure Osmometer 5600, Vapro®) and titer (IMMAGE 800 Protein Chemistry Analyzer, Beckman Coulter) measurements. On day 14, the culture was harvested and centrifuged (4000 rpm, 45 min, 4 °C) to collect the supernatant for downstream processing.

### AKTA chromatography

MabSelect™ PrismA (Cytiva) resin was packed into a XK16™ column (Cytiva), reaching a bed height of 12 cm (CV ~ 24.1 mL). This column was then connected to an AKTA™ Avant 25 (Cytiva) for the purification process.

Purification was performed using an intermediate low pH wash condition, optimized for Fab-scFv format bsAb (Chen et al. 2022). In brief, the column was equilibrated with 100 mM sodium phosphate and 150 mM NaCl at pH 7.2 prior to loading harvest cell culture fluid (HCCF) (< 31.5 mg of monomeric bsAb/mL resin). Following the loading step, the column underwent a 3-column volume (CV) wash with 50 mM Na-citrate at pH 6.0, succeeded by a 10 CV wash with 50 mM Na-citrate at pH 4.7. The elution of bsAb was achieved using 50 mM sodium citrate at pH 3.6, and subsequent neutralization was performed with 1.0 M Tris at pH 8.0, reaching a final product pH of 6.5. The pH values of the collected eluate and the neutralized product were measured, if necessary, using an external pH probe (Mettler Toledo). The neutralized product was filtered (0.22 µm), prior to use for the study. This post-filtration material is referred to as post-ProA and is used as the material for the entire study.

### Preparation of bsAb A samples to study environmental influences

#### pH effect

A post-Protein A-purified bsAb A (pH 6.5) underwent buffer exchange into McIlvaine buffers pH 3.5, 5.5, and 7.5, respectively. Protein purity was subsequently monitored over a 7-day period at room temperature (25 ± 1 °C) to assess protein stability at different pH conditions. These three pH values (pH 3.5, 5.5, and 7.5) were selected because they are commonly used during purification and formulation processes. The McIlvaine buffers, with their broad pH capacity (pH 2.2–8), were employed to minimize variability arising from different buffer types.

#### Buffer effect

Post-Protein A-purified bsAb A (pH 6.5) underwent buffer exchange into four buffer recipes without salt at pH 5.5: 50 mM sodium acetate (NaOAc), 50 mM sodium citrate (NaCi), 50 mM sodium phosphate (NaPi), and 50 mM histidine-HCl (His-HCl). These buffers were selected as they are common buffer types, used during purification/formulation processes. The samples were assessed for purity influenced by buffer at room temperature (25 ± 1 °C) over 1 month using SEC-MALS.

#### Ionic strength effect

Post-Protein A-purified bsAb A (pH 6.5) was buffer exchanged into 50 mM histidine-HCl buffer at pH 5.5, containing 0, 150, or 500 mM NaCl, or 150 mM arginine-HCl. Samples were incubated at room temperature (25 ± 1 °C), and the purity was tracked over 1 month using SEC-MALS.

#### Protein concentration effect

Post-Protein A-purified bsAb A (pH 6.5) was buffer exchanged into 50 mM histidine-HCl buffer at pH 5.5 at final total protein concentrations of 1 and 10 mg/mL, prior to incubation at room temperature (25 ± 1 °C). The sample purity was monitored over 1 month using SEC-MALS.

#### Temperature effect

Post-Protein A-purified bsAb A (pH 6.5) underwent buffer exchange into 50 mM histidine-HCl buffer at pH 5.5 at a final protein concentration of 1 mg/mL. The samples were then incubated at room temperature (25 ± 1 °C) and 40 ± 1 °C. The sample purity was monitored over 1 month using SEC-MALS. We chose to track bsAb A purity at room temperature as it is a typical temperature during the manufacturing processes, while 40 °C is commonly used for accelerated stability studies and could be accidentally reached during therapeutic transportation.

### BsAb purity and molecular size determination using SEC-MALS

Purity, concentration, and molecular size of bsAb were assessed via size exclusion chromatography with multi-angle static light scattering (SEC-MALS). Samples, prepared at 0.25 mg/mL, were injected into the SEC-MALS for analysis (100 µL per injection). Employing UHPLC with a variable wavelength UV detector set at 280 nm (Thermo Fischer Scientific, Waltham, MA), separation occurred using a TSKgel G3000SWXL column (7.8 mm i.d. × 30 cm; Tosoh Bioscience) at a flow rate of 0.6 mL/min. The mobile phase, 200 mM l-arginine, 50 mM MES, 5 mM EDTA, 0.05% sodium azide (w/w), pH 6.5, was filtered through Durapore, PVDF 0.1 mm membrane filters (Merck Millipore) prior to use. Post-column, bsAb concentration was gauged with a UV detector (A280) by integrating the peak area of the chromatographic main peak and referencing to a calibration curve established with an antibody standard with known concentration (Chen et al. 2022). The relative peak areas (A280) were used to assess bsAb purity while the molecular sizes were determined via MALS (Wyatt Technology, Santa Barbara, CA). The latter utilized ASTRA software V 8.1.2 (Wyatt Technology) for data collection and processing.

## Results

Our study investigated the impact of pH, buffer types, ionic strength, protein concentration, and temperature on the stability of Fab-scFv format bsAb A, employing SEC-MALS to track sample purity (%monomer) and monomer concentration over time. Monomeric bsAb A appeared as the main peak (retention time = 13.2–16.4 min), with dimers (retention time = 11.7–13.2 min) and larger heterogeneous aggregates (retention time < 11.7 min) (Supplementary Fig. 1). Protein purity analysis considered both dimers and larger aggregates when calculating the percentage of high molecular weight species (%HMW).

The results demonstrate that under certain conditions, purity of bsAb A increased, accompanied by observable increases in monomer concentrations over time, suggesting the reversal of self-associated aggregates into monomers (also known as reversible self-association, RSA). To assess the reversal of self-associated aggregates of bsAb A to monomers, we introduced a term ‘Relative Monomer Concentration (RMC)’ (Eq. 1), measuring monomer concentration relative to its initial concentration. RMC > 1 indicates the occurrence of the reversal of self-associated aggregates to monomers, while RMC < 1 suggests less likelihood for the reversal.

### pH effect

**Fig. 2:**
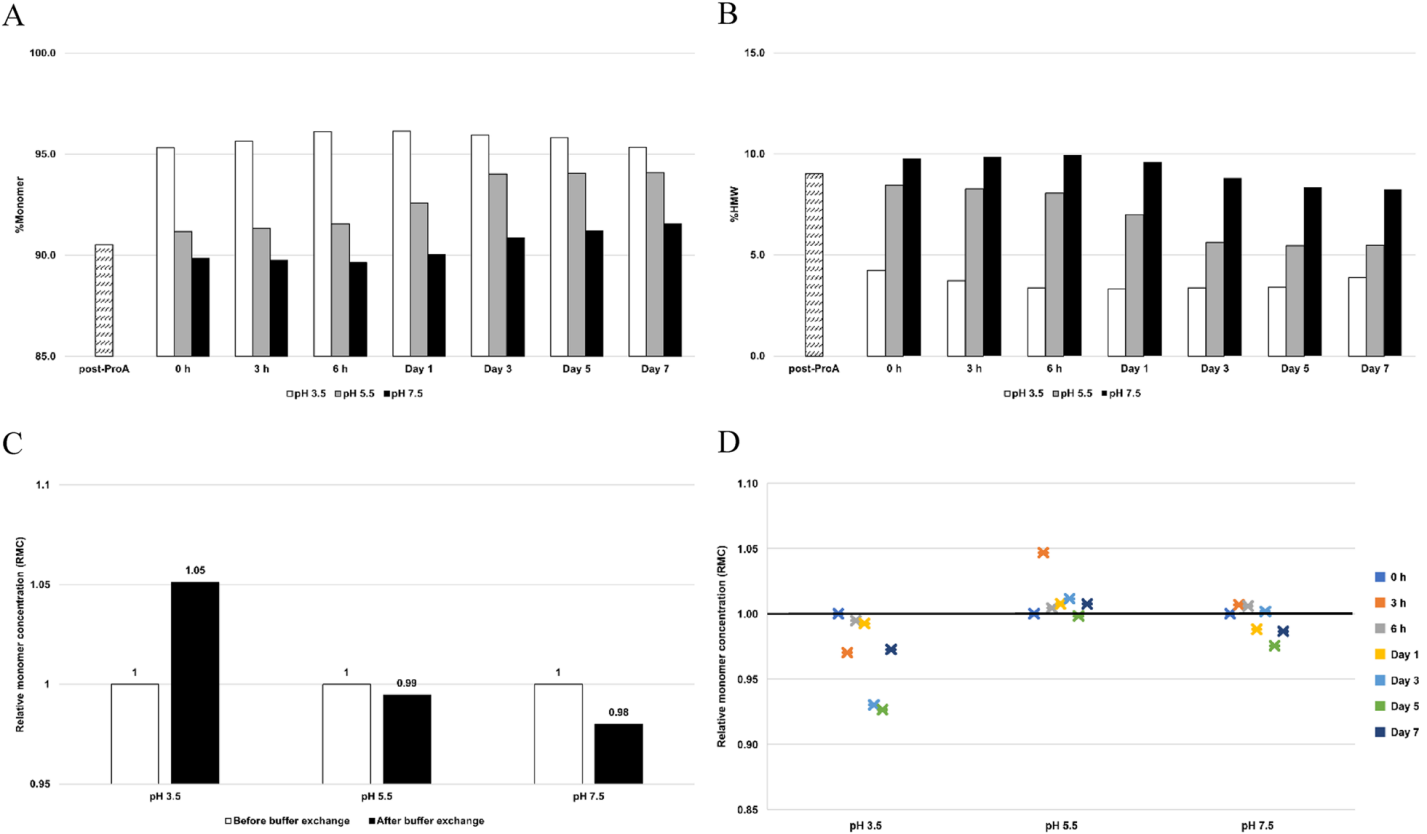
Analysis of purity and relative monomer concentration (RMC) for bsAb A at a total protein concentration of 1 mg/mL during 7-day incubation at room temperature at different pH conditions. **A** %Monomer and **B** %HMW. The post-ProA refers to Protein A eluate at pH 6.5, and 0 h refers to post-ProA buffer-exchanged into McIlvaine buffers at the indicated pH before incubation. **C** Monomer concentration at 0 h relative to its initial concentration at post-ProA; and **D** monomer concentration post incubation relative to its initial concentration at 0 h. A black horizontal line represents RMC = 1, indicating maintained monomer concentration. RMC > 1 indicates the occurrence of the reversal of self-associated aggregates to monomers, while RMC < 1 suggests less likelihood for the reversal

Distinct purity profiles of bsAb A were observed at pH 3.5, 5.5, and 7.5 at ‘0 h’ after buffer exchange. Shifting the protein from its original pH of 6.5 to pH 3.5 led to a marked increase in %monomer (from 90.5% to 95.3%) (Fig. 2A, Supplementary Fig. 1) with a reduction in %HMW (from 9.0 to 4.2%) (Fig. 2B). SEC-MALS analysis demonstrated the decrease in a dimer peak, together with an increase in the monomeric peak (Supplementary Fig. 1). Furthermore, abrupt change to low pH of 3.5 provided an immediate effect on the reversal of self-associated aggregates to monomer as indicated by observable monomer concentration increase (RMC = 1.05) (Fig. 2C).

Conversely, lower RMC values were observed at higher pH conditions. Buffer exchange to achieve the final pH of 5.5 resulted in a slight increase in %monomer (90.5% to 91.2%) (Fig. 2A) and a decrease in %HMW (9.0% to 8.5%) (Fig. 2B), with relatively maintained monomer concentration (RMC = 0.99) (Fig. 2C). Exchanging bsAb A to a final pH of 7.5 resulted in a slight decrease in %monomer (90.5% to 89.9%) (Fig. 2A), an increase in %HMW (9.0% to 9.8%) (Fig. 2B), and slight decrease in monomer concentration (RMC = 0.98) (Fig. 2C). The results suggested that reversal of self-association was less likely when the pH is higher.

Different purity profiles post-buffer exchange to varying pH values may be explained by the net charge on the protein surface. When bsAb A shifted from pH 6.5 to pH 3.5, its net positive charge significantly intensified, resulting in a marked increase in strong repulsive electrostatic forces among the molecules (Gentiluomo et al. 2020; Andersen et al. 2010; Tian et al. 2014). This repulsion can literally prevent short-range interactions, as described by the proximity energy theory (Laue 2012). Therefore, a sudden shift in pH facilitated an immediate enhancement in protein purity, along with monomer concentration increase (Fig. 2).

Conversely, fewer changes observed in %monomer at the final buffer pH of 5.5 could be attributed to the lower positive net charge on the protein surface, leading to reduced repulsion. Consequently, one might expect diminished improvement in purity, as well as the reversal of self-associated aggregates comparing pH 3.5. On the other hand, the buffer at pH 7.5 provides an environment in which the pH approaches a pI of bsAb A (pI = 8.52), further neutralizing the net charge of bsAb A. This lessens the electrostatic effect, while placing more weight on hydrophobic forces, potentially triggering aggregation formation.

**Fig. 3:**
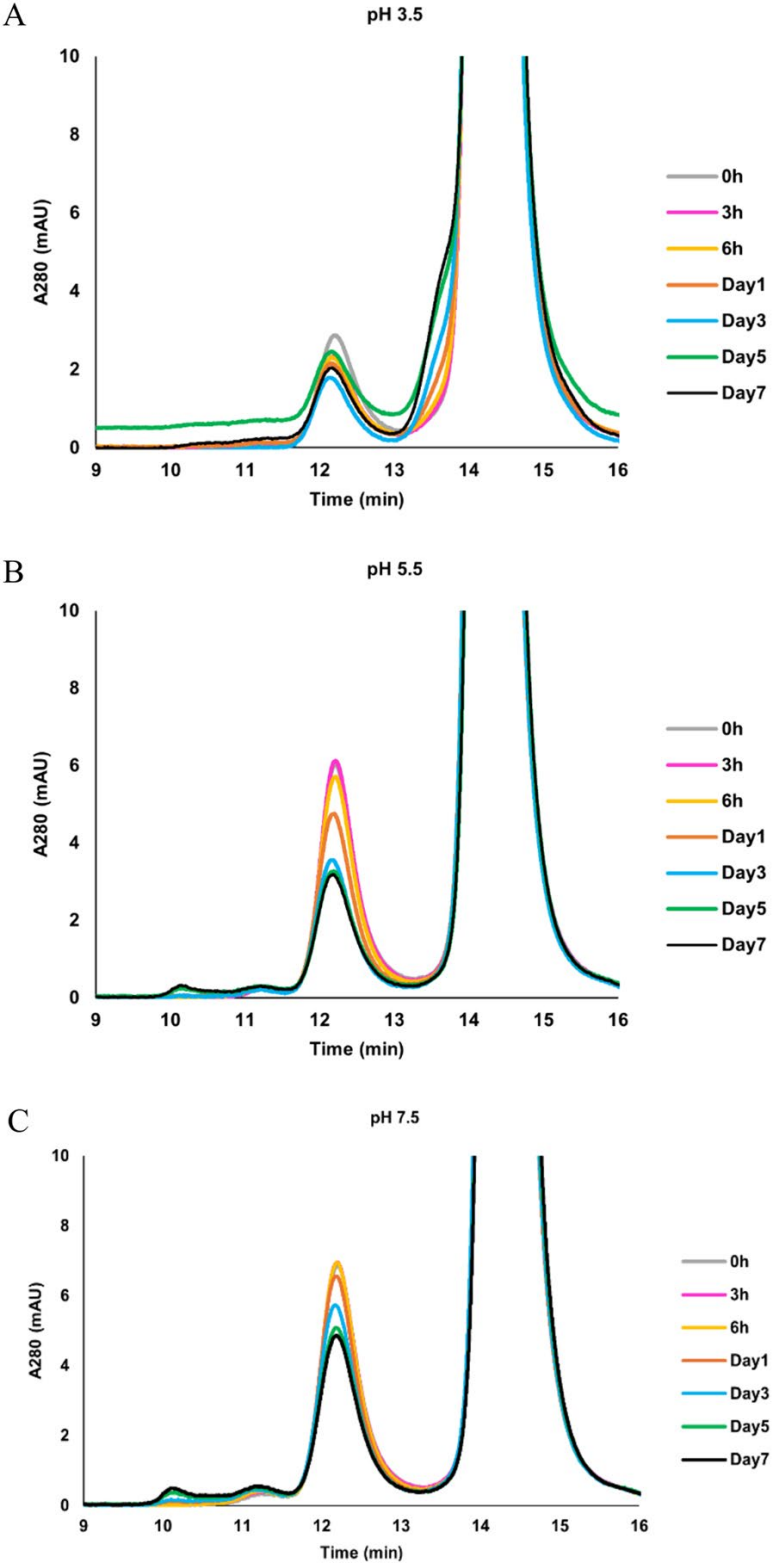
Overlay of bsAb A SEC-MALS profiles during a room temperature incubation period from 0 h to Day 7 at different pH conditions: **A** pH 3.5, **B** pH 5.5, and **C** pH 7.5. The appearance of frontal broadening in the peak at pH 3.5 suggests potential structural fluctuation and/or partial protein unfolding over time

After the 7-day incubation period, monomeric bsAb A persisted as the predominant population in protein solutions at all tested pH conditions (Fig. 3). However, at pH 3.5, we observed limited improvement in protein purity during the first day of incubation (%monomer: 95.3% to 96.1%) (Fig. 2A), followed by a slight decline in purity thereafter [%monomer: 96.1% (Day 1) to 95.3% (Day 7)]. Furthermore, a decrease in monomer concentration was detected over time, with a distinct decline after day 1 (Fig. 2D), suggesting no evidence of the reversal of self-associated aggregates to monomers at the low pH of 3.5. Additionally, SEC-MALS profiles revealed an increase in the frontal broadening of the monomeric peak after 1 day of incubation at pH 3.5 (Fig. 3A), contrasting with the absence of such broadening at pH 5.5 (Fig. 3B) and pH 7.5 (Fig. 3C). This observation suggests the possibility of structural fluctuation and/or partial protein unfolding at pH 3.5, which is less likely to occur when bsAb A is at pH 5.5 and pH 7.5.

At pH 5.5, the purity of bsAb A improved with an increase in %monomer [91.2% (Day 1) to 94.1% (Day 7)] (Fig. 2A), accompanied by a decrease in %HMW (8.5% to 5.5%) (Fig. 2B) during the 7 day-incubation. Slight increase in monomer concentration was also observed (Fig. 2D), suggesting the reversal of self-associated species. In contrast, a slight decrease in bsAb A purity was observed during the initial 6 h of incubation at pH 7.5 (%monomer: 89.9% to 89.6%) (Fig. 2A), with a slightly higher percentage of HMW (%HMW: 9.8% to 10%) (Fig. 2B). Subsequently, a slight increase in %monomer was observed after 6 h of incubation [%monomer: 89.6% (6 h) to 91.6% (day 7)], accompanied by a decrease in HMW [10% (6 h) to 8.2% (day 7)]. Despite slight improvement in purity after 6-h incubation, a loss in monomer was detected instead (Fig. 2D). Additionally, the observation of larger HMW (retention time < 11.7 min) was most pronounced at pH 7.5 after a 7-day incubation compared to the other two pH conditions (Fig. 3, Supplementary Fig. 2). These findings suggest that the stability of bsAb A was compromised at the two extreme pH values (pH 3.5 and 7.5), while stability was most sustained at the slightly acidic pH of 5.5. Given that pH 5.5 exhibited the best purity retention, we selected this pH value for further investigation.

### Buffer effect

**Fig. 4:**
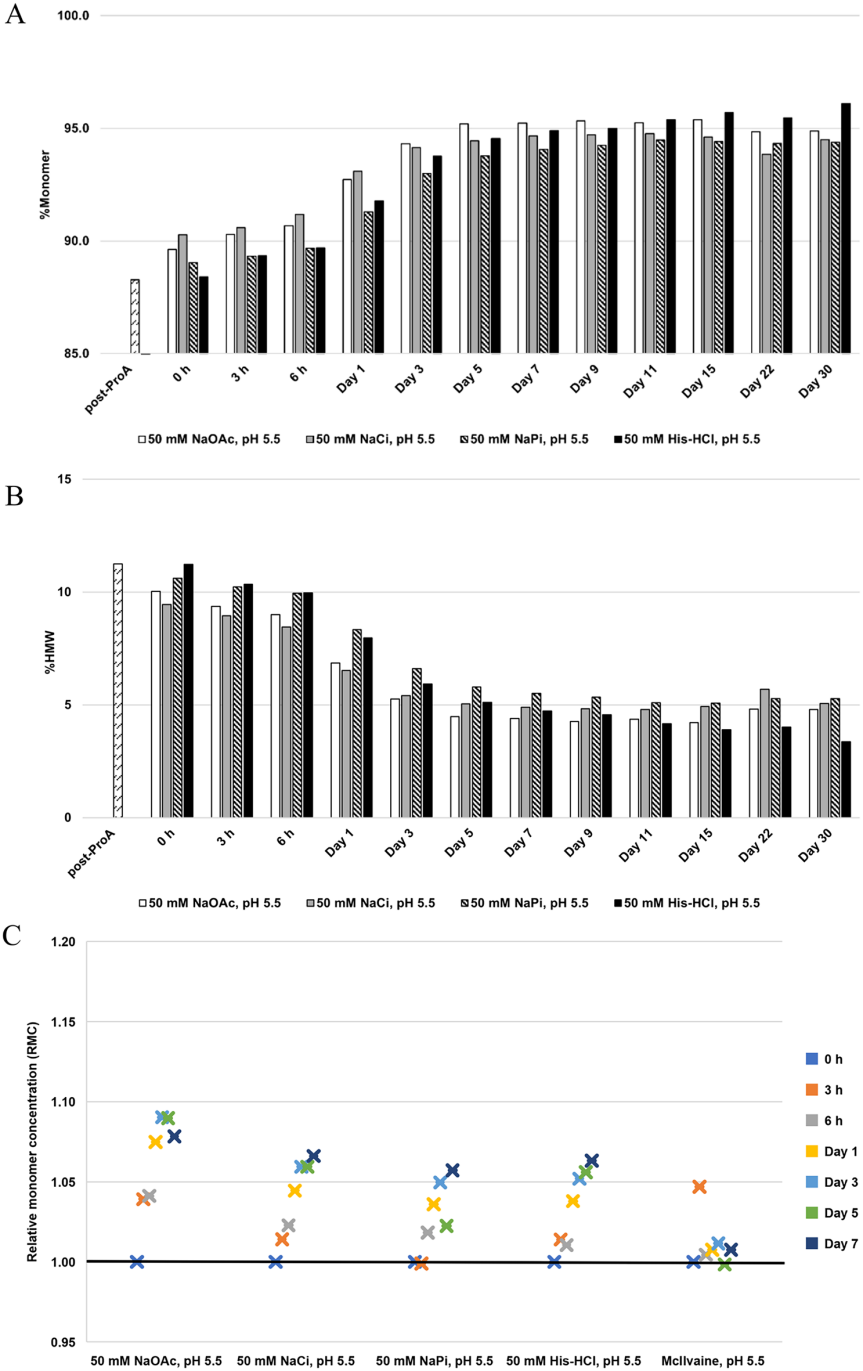
Analysis of purity and relative monomer concentration (RMC) for bsAb A at a total protein concentration of 1 mg/mL during 30-day incubation at room temperature in different buffers. **A** %Monomer and **B** %HMW. The post-ProA refers to Protein A eluate at pH 6.5, and 0 h refers to post-ProA buffer-exchanged into indicated buffers before incubation. **C** Monomer concentration post incubation relative to its initial concentration at 0 h. A black horizontal line represents RMC = 1, indicating maintained monomer concentration. RMC > 1 indicates the occurrence of the reversal of self-associated aggregates to monomers, while RMC < 1 suggests less likelihood for the reversal

The results suggested that bsAb A is stable in all tested buffer recipes, with %monomer improving from 88.4–90.3% to 94.4–96.1% (Fig. 4A), together with reduction of %HMW from 9.5–11.2% to 3.4–5.3% (Fig. 4B) over 1 month. Interestingly, while the four buffers demonstrate a similar trend of increased monomer concentrations over the 7-day incubation period, the monomer concentrations remained relatively constant in McIlvaine pH 5.5 (Fig. 4C).

As histidine has been widely used to formulate antibodies (Baek et al. 2019; Saurabh et al. 2022), 50 mM histidine-HCl pH 5.5 was selected as a foundational buffer to further investigate impacts from other environmental factors on bsAb A stability. The preference for the histidine buffer on protein stabilization also aligns with previous studies, indicating that 10 mM histidine at pH 5.5–6.5 has minimal impact to trigger protein aggregate formation in the selected monoclonal antibody, thereby maintaining protein purity/stability (Esfandiary et al. 2015).

### Ionic strength

**Fig. 5:**
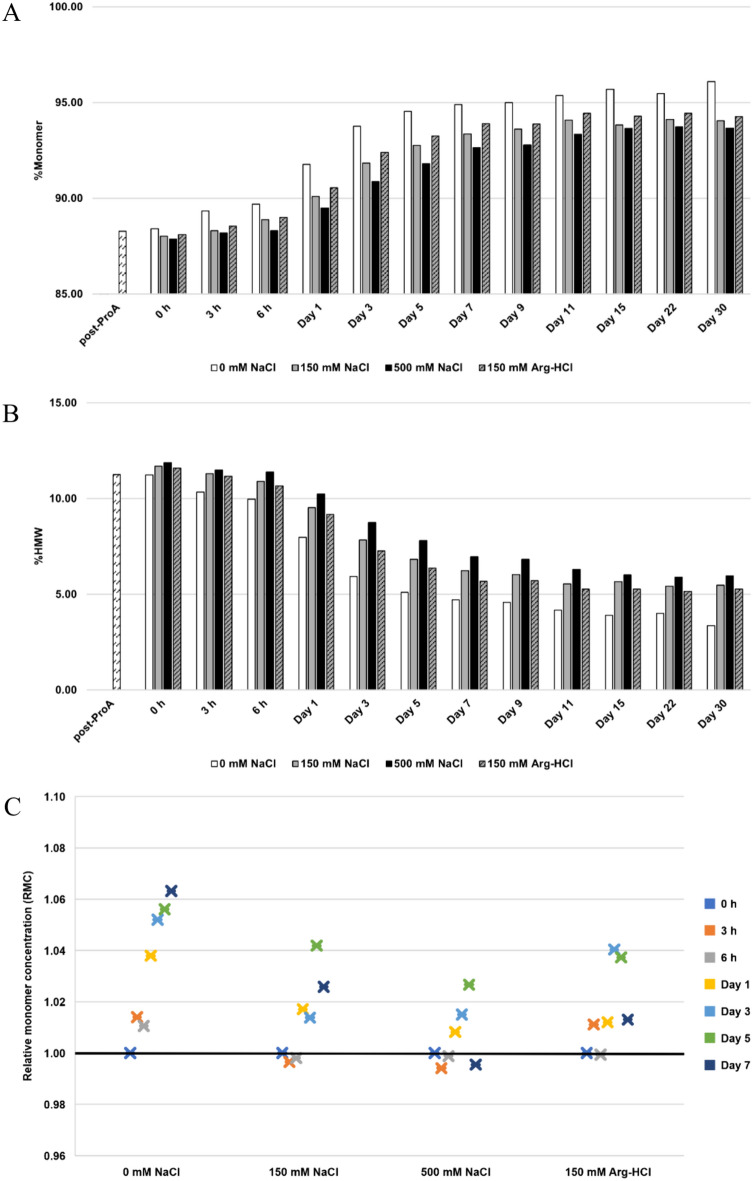
Analysis of purity and relative monomer concentration (RMC) for bsAb A at a total protein concentration of 1 mg/mL during 30-day incubation at room temperature in 50 mM histidine-HCl, pH 5.5 with different salt concentrations. **A** %Monomer and **B** %HMW. The post-ProA refers to Protein A eluate at pH 6.5, and 0 h refers to post-ProA buffer-exchanged into indicated buffers before incubation. **C** Monomer concentration post incubation relative to its initial concentration at 0 h. A black horizontal line represents RMC = 1, indicating maintained monomer concentration. RMC > 1 indicates the occurrence of the reversal of self-associated aggregates to monomers, while RMC < 1 suggests less likelihood for the reversal

Overall, our results demonstrate that bsAb A remained stable in 50 mM histidine-HCl across a range of salt concentrations tested (0–500 mM NaCl) (Fig. 5A). Interestingly, we observed an improvement in protein purity, with the solution of minimal ionic strength facilitating this enhancement. Over 30 days, buffer with 0 mM NaCl exhibited the most rapid improvement in protein purity compared to buffers with higher NaCl concentrations (% monomer: from 88.4% to 96.1% (0 mM NaCl); from 88.0 to 94.1% (150 mM NaCl); and from 87.9 to 93.7% [500 mM NaCl)] (Fig. 5A). Further analysis of the impact of different salts on protein stability revealed comparable levels of stability for bsAb A in 50 mM histidine-HCl pH 5.5, regardless of whether it contained 150 mM sodium chloride (%monomer from 88.0 to 94.1%) or 150 mM arginine-HCl (%monomer from 88.1 to 94.3%).

Enhancement in purity traces was observed, together with detectable reversal of bsAb A self-associated aggregates to monomers over 7 days under all tested salt conditions (Fig. 5C). The RMC plot indicates that the absence of sodium chloride (0 mM NaCl) notably accelerated the restoration of monomers from self-associated aggregates, while higher sodium chloride concentrations (150 and 500 mM) slowed down this process. Moreover, both types of salts (NaCl and Arg-HCl) at the same concentration (150 mM) demonstrated similar levels of RMC. As 50 mM histidine-HCl pH 5.5 in the absence of NaCl was the most efficient condition for maintaining bsAb A stability, this buffer condition was selected for further experimentation.

### Protein concentration effect

**Fig. 6:**
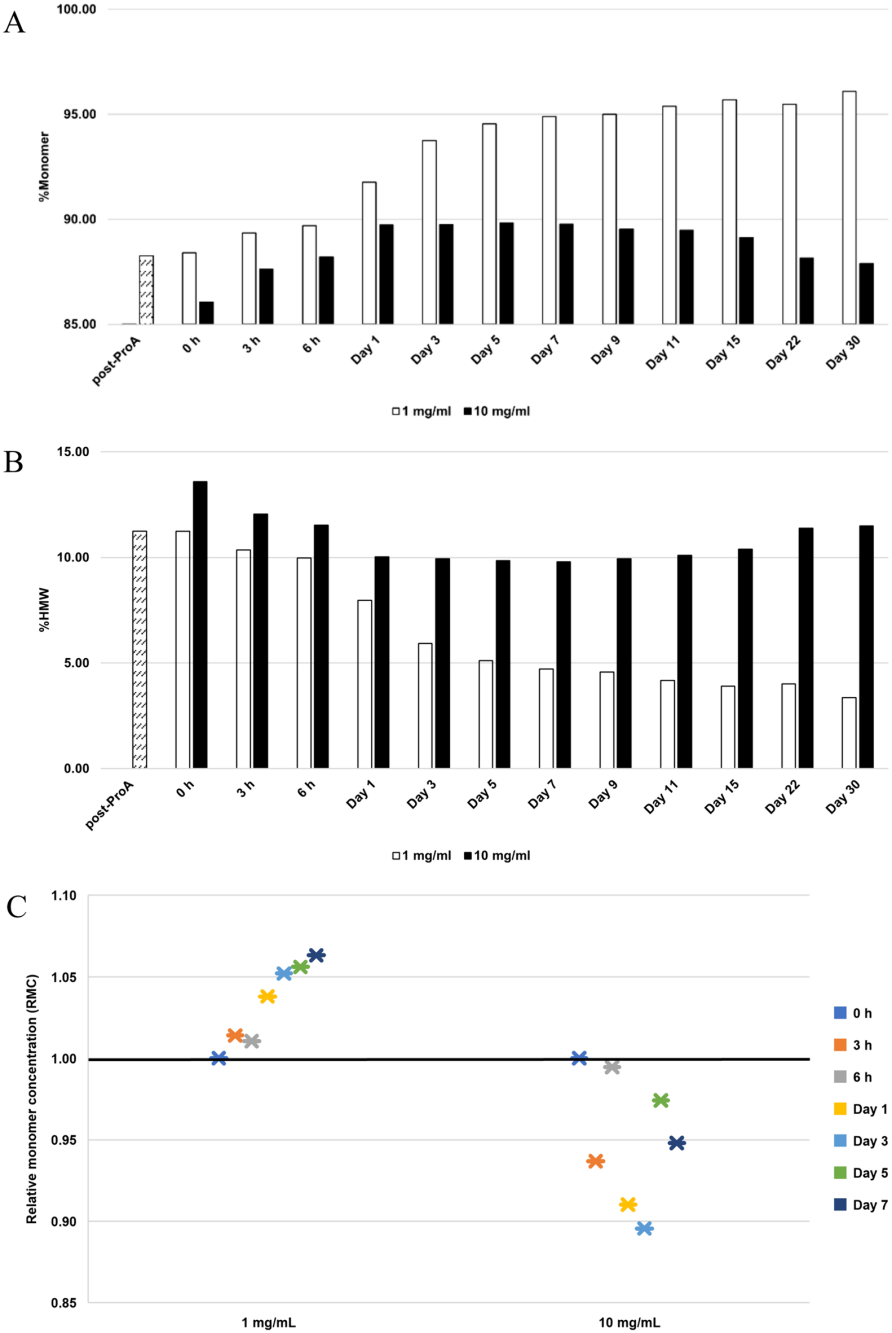
Analysis of purity and relative monomer concentration (RMC) for bsAb A during 30-day incubation at room temperature in 50 mM histidine-HCl, pH 5.5 with different protein concentrations. **A** %Monomer and **B** %HMW. The post-ProA refers to Protein A eluate at pH 6.5, and 0 h refers to post-ProA buffer-exchanged into 50 mM histidine-HCl, pH 5.5 at indicated protein concentrations before incubation. **C** Monomer concentration post incubation relative to its initial concentration at 0 h. A black horizontal line represents RMC = 1, indicating maintained monomer concentration. RMC > 1 indicates the occurrence of the reversal of self-associated aggregates to monomers, while RMC < 1 suggests less likelihood for the reversal

At a lower protein concentration of 1 mg/mL, a significant improvement in bsAb A purity was observable during the 7-day incubation period. The %monomer increased from 88.4% (0 h) to 94.9% (Day 7) (Fig. 6A), accompanied by a decrease in high molecular weight species (%HMW) from 11.2% (0 h) to 4.7% (Day 7) (Fig. 6B) and detectable monomer concentration increase (Fig. 6C). Subsequently, the purity was relatively maintained over the course of 1 month, with the %monomer ranging from 94.9% (Day 7) to 96.1% (Day 30) (Fig. 6A).

However, at a higher protein concentration of 10 mg/mL, the observed purity enhancement was limited, with only a 3.7% increase in %monomer observed from 86.1% (0 h) to 89.8% (Day 5) (Fig. 6A). Concurrently, there was a decrease in %HMW from 13.6% (0 h) to 9.9% (Day 5) (Fig. 6B), along with evidence of monomer loss (Fig. 6C). Subsequently, a detectable decline in purity was noted from day 7 onwards, with %monomer decreasing from 89.8% (Day 7) to 87.9% (Day 30) (Fig. 6A), accompanied by an increase in %HMW from 9.8% (Day 7) to 11.5% (Day 30) (Fig. 6B). The evidence suggests that bsAb A stability was compromised at a high concentration of 10 mg/mL in 50 mM histidine-HCl, pH 5.5, with no indication of reversible self-association.

### Temperature effect

**Fig. 7:**
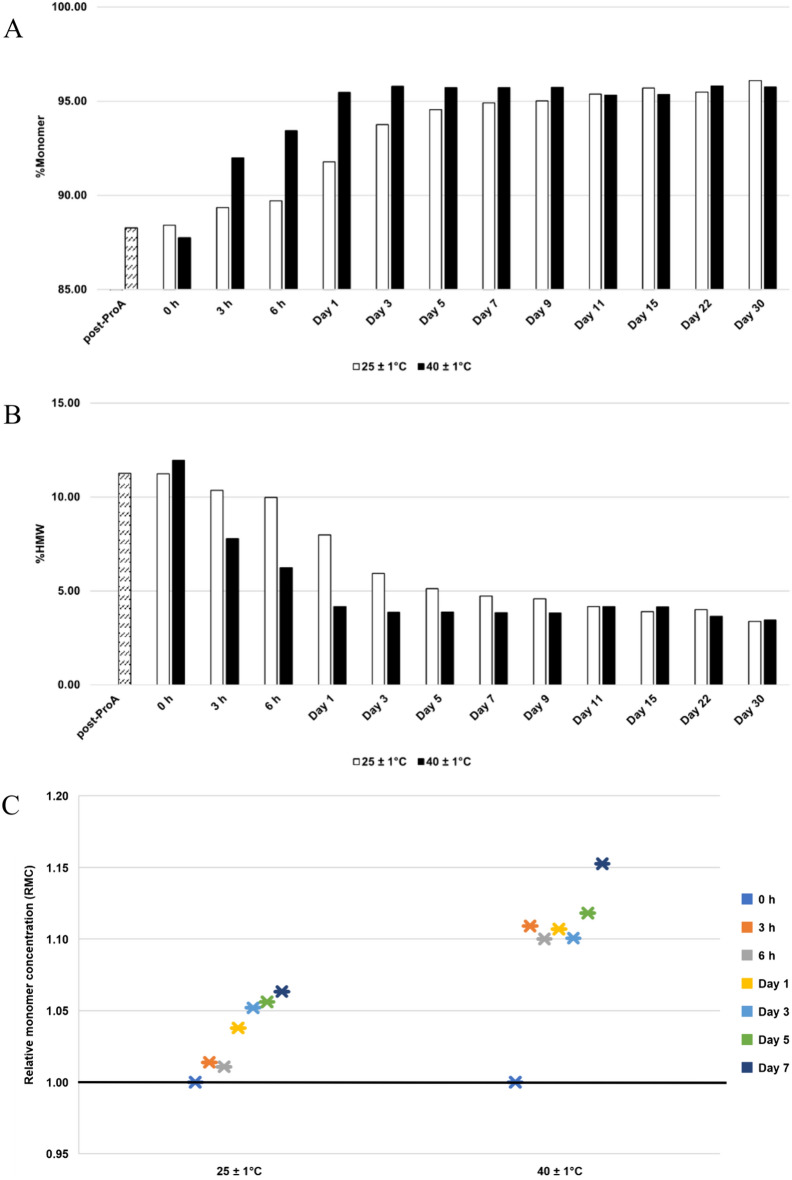
Analysis of purity and relative monomer concentration (RMC) for bsAb A at a total protein concentration of 1 mg/mL during 30-day incubation in 50 mM histidine-HCl, pH 5.5 with different temperatures. **A** %Monomer and **B** %HMW. The post-ProA refers to Protein A eluate at pH 6.5, and 0 h refers to post-ProA buffer-exchanged into 50 mM histidine-HCl, pH 5.5 at indicated temperature before incubation. **C** Monomer concentration post incubation relative to its initial concentration at 0 h. A black horizontal line represents RMC = 1, indicating maintained monomer concentration. RMC > 1 indicates the occurrence of the reversal of self-associated aggregates to monomers, while RMC < 1 suggests less likelihood for the reversal

Remarkably, bsAb A demonstrated stability under both temperature conditions over the course of a month, yielding the final purity of 96.1% at room temperature and 95.7% at 40 °C, respectively (Fig. 7A). This observation underscores the robust stability of bsAb A at a concentration of 1 mg/mL, even under the heightened temperature of 40 °C.

Furthermore, the elevated temperature of 40 °C notably enhanced bsAb A purity during the initial 3-day incubation, evidenced by an increase in %monomer from 87.7% (0 h) to 95.8% (Day 3) (Fig. 7A), accompanied by a reduction in %HMW from 12% (0 h) to 3.9% (Day 3) (Fig. 7B) and pronounced monomer concentration increase (Fig. 7C), indicating an occurrence of rapid reversible self-association. Subsequently, both %monomer (Fig. 7A) and %HMW (Fig. 7B) stabilized from Day 3 to Day 30, suggesting sustained protein stability.

While high temperature facilitated an enhancement in bsAb A purity, purity improvement was also observed at room temperature, albeit at a slower rate compared to the 40 °C incubation. Over the same initial 3-day incubation, %monomer increased from 88.4% (0 h) to 93.8% (Day 3) (Fig. 7A), with %HMW decreasing from 11.2% (0 h) to 5.9% (Day 3) (Fig. 7B). Furthermore, monomer concentration increase was detected, albeit to a lesser extent than during incubation at 40 °C (Fig. 7C). These results indicate the resilience of bsAb A stability even at the elevated temperature of 40 °C, which also expedited the reversal of self-associated species into their monomeric state.

## Discussion

To better understand the impact of environmental factors on the stability of Fab-scFv format bsAb, a comprehensive understanding of molecular mechanisms governing protein aggregation formation is crucial as protein aggregation diminishes protein stability. These mechanisms encompass an energy landscape associated with protein folding and misfolding, along with grasping the molecular events (or steps) occurring during the aggregation processes (Li, et al. 2016; Pang et al. 2023).

Using antibody aggregation mechanisms as an example, the energy landscape pertains to thermodynamically driven events (Clarkson et al. 2016). The interplay of enthalpy and entropy guides either protein folding or misfolding towards the formation of the most thermodynamically stable species, often referred to as a free-energy minimum (Li, et al. 2016; Pang et al. 2023). Under favorable conditions, these factors drive protein folding towards the formation of native monomers as the free-energy minimum species. Conversely, under certain undesirable conditions, protein misfolding may occur instead, leading to the addition of aggregates, being introduced as more local minima in the free energy landscape (Dobson et al. 1998; Knowles et al. 2014).

Concerning the molecular events for aggregation formation, one visualizes that protein monomers and various states of self-associated aggregates in solution coexist in equilibrium (Clarkson et al. 2016; Roberts 2007). Understanding the molecular events involves deciphering the transformations among these antibody populations, and how the equilibrium is altered under different environmental conditions.

**Fig. 8:**
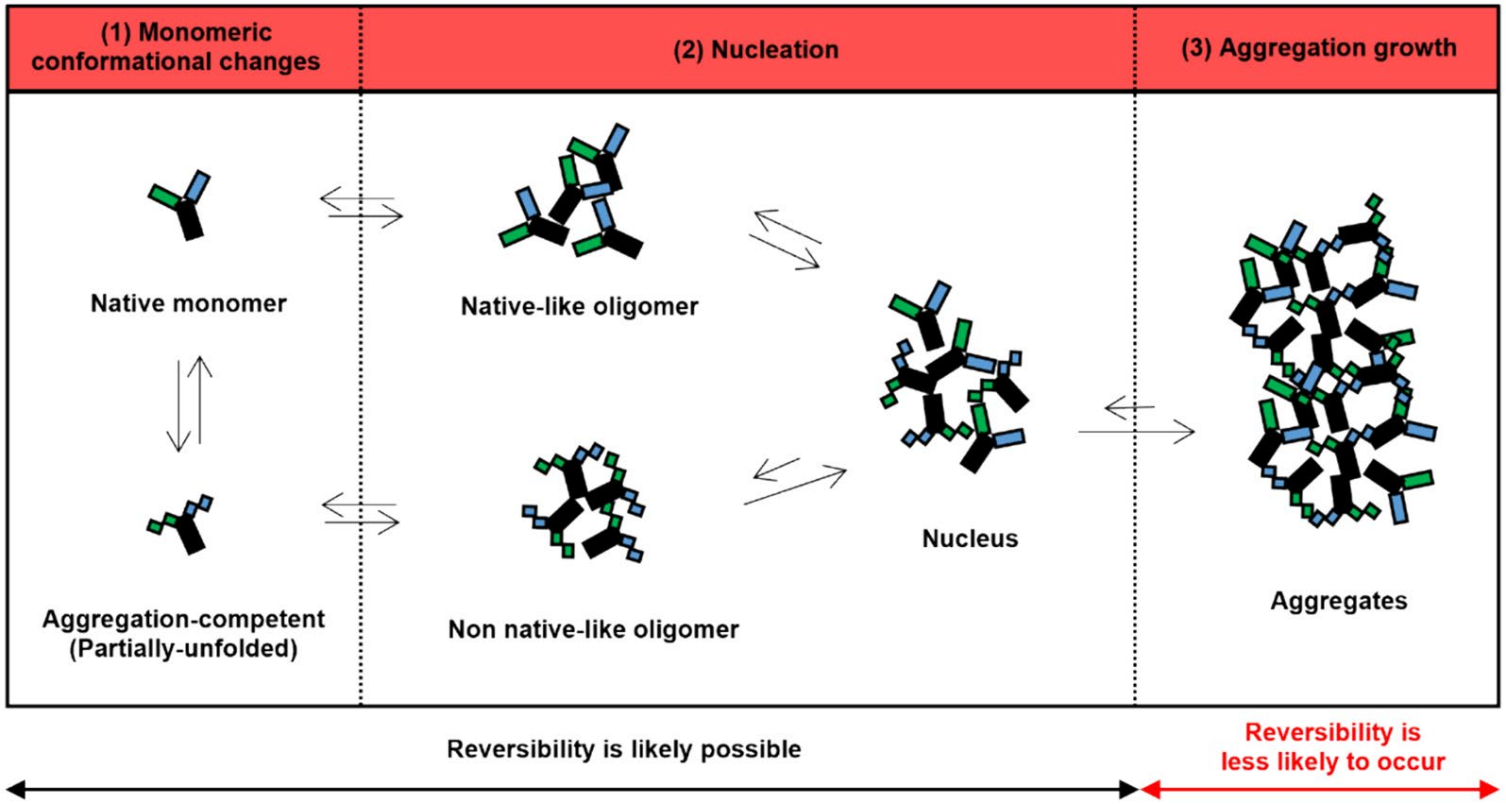
Simplified molecular mechanisms of antibody aggregate formation, comprising three key steps: (1) monomeric conformational changes, (2) nucleation, and (3) aggregation growth. Adapted from Li et al. (2016), Pang et al. (2023). Steps (1) and (2) still have the potential to be reversible, while step (3) is more likely to be irreversible

Figure 8 depicts the molecular events of antibody aggregation, encompassing three key steps that occur dynamically. A pivotal initial event preceding self-assembly occurs when native monomers undergo “monomeric conformational changes” (or partially unfolding) to form the “aggregation-competent” state (Li, et al. 2016; Pang et al. 2023; Roberts 2007; Andrews and Roberts 2007). This transformation may involve either structural fluctuation or partial unfolding, generating intermediate states or partially unfolded species.

Subsequently, nucleation occurs as the second step, wherein the intermediates self-assemble to form oligomers (Fig. 8, Step 2). The aggregation-competent entities often engage in self-association, leading to the creation of “non-native like oligomers,” generally exhibiting a high propensity for further aggregation events. Despite this, it is noteworthy that native monomers themselves can undergo “self-assembly” to produce “native-like oligomers,” capable of reverting to monomers through the mechanism of “reversible-self association (RSA)”. While the reversibility of oligomers also applies to non-native like oligomers during the nucleation event, it tends to occur to a lesser extent than with native-like oligomers. Consequently, the equilibrium leans towards additional aggregation events, rather than the reversal of the self-assembly (Chi et al. 2003; Wang 2005).

After nucleation, both types of oligomers undergo additional aggregation growth events, where either monomers or aggregates can be incorporated into the existing aggregates, leading to the formation of larger aggregates (Fig. 8, Step 3). Although the theoretical reversibility of larger aggregates to monomers at this stage may be possible, it is highly improbable due to the forward direction (aggregation formation) occurring at significantly higher rates (Cohen, et al. 2011).

Our study illustrates that the stability of bsAb A can be effectively elucidated by considering the molecular events involved in aggregation formation, as described earlier.

### Differential aggregation mechanisms underlying bsAb A stability at the two extreme pH conditions

Our findings suggest that the stability of bsAb A is compromised over 7 days under both low pH condition (pH 3.5), and the pH, approaching an isoelectric point (pI) of bsAb A (pI = 8.52) (pH 7.5), potentially through distinct aggregation mechanisms.

At pH 3.5, the strong positive charge on the protein surface causes immediate molecular repulsion, resulting in a notable increase in %monomers shortly after buffer exchange from pH 6.5 to pH 3.5 (Fig. 2A). Additionally, observable monomer concentration increase is evident (Fig. 2C). However, prolonged exposure to low pH destabilizes the protein, as indicated by the emergence of a broadening monomeric peak on SEC-MALS after a 1-day incubation period (Fig. 3A). This suggests potential monomeric structural fluctuations and/or partial protein unfolding, which may generate aggregation-competent species, thereby favoring further aggregation events rather than the reversal of self-associated species (Fig. 8, Step 1). The reduction in %monomer observed after 1 day of incubation at pH 3.5, accompanied by monomer loss, further supports this assertion (Fig. 2). It is noteworthy that the susceptibility to structural fluctuation and/or protein unfolding is highly dependent on intrinsic properties of particular proteins. With the same duration of low pH exposure, some proteins may experience partial unfolding, while others may undergo acid-induced oligomerization, either reversibly or irreversibly (Skamris et al. 2016).

Our findings align with previous studies indicating that extremely low pH can induce changes in protein tertiary structures, initiating partial unfolding and the creation of aggregation-competent states (Gentiluomo et al. 2020; Esfandiary et al. 2015). Additionally, structural flexibility of the protein plays a crucial role. Proteins with high structural flexibility tend to expose their electrostatic surfaces more to interact with the surrounding aqueous solvent while concealing hydrophobic portions, thereby optimizing electrostatic repulsion, and preserving protein solubility (Xu et al. 2023). However, low pH conditions notably decrease protein flexibility, therefore triggering protein unfolding (Xu et al. 2023). In this scenario, the equilibrium between monomers and aggregates shifts toward aggregation formation as it becomes more thermodynamically favourable. In other words, protein stability is compromised at low pH, which explains the instability of bsAb A at pH 3.5.

However, aggregation formation likely follows a different pathway at pH 7.5, nearing the isoelectric point (pI) of bsAb A (pI = 8.52). Here, hydrophobic forces are anticipated to outweigh electrostatic contributions. This is evidenced by a slight drop in bsAb A purity (Fig. 2A), and higher %HMW observed during the initial 6 h of incubation (Fig. 2B). Subsequently, although the %monomer slightly increased (Fig. 2A), and %HMW decreased (Fig. 2B), monomer loss was observed (Fig. 2C), indicating that some portions of bsAb A in solution likely transitioned into irreversible aggregates that cannot be detected by SEC-MALS. Furthermore, the absence of a broadened peak on SEC-MALS (Fig. 3C), along with the presence of significant high molecular weight components at pH 7.5 after a 7-day incubation period, compared to pH 3.5 and pH 5.5, suggests that aggregation at pH 7.5 is probably governed by hydrophobic effects, unlike at pH 3.5, which likely involves electrostatic interactions (Fig. 3, Supplementary Fig. 2). At both extreme pH values, aggregation appears to occur irreversibly, as demonstrated by monomer loss over time (7 days). However, it is noteworthy that reversibility may be possible upon neutralization (Skamris et al. 2016). However, the later was beyond the scope of this study.

Evidently, pH 5.5 emerges as an optimal condition for maintaining the stability of bsAb A, as indicated by the sustained %monomer (Fig. 2A) and consistent monomer concentrations (Fig. 2D) observed after day 3 of incubation. At this pH, the protein maintains a slight positive net charge, fostering repulsive interactions while potentially promoting protein structural flexibility, thereby contributing to its stability compared to pH 3.5. Consequently, correct protein folding is more likely to be thermodynamically favoured over misfolding, thereby maintaining the monomeric state as the local free-energy minimum. In essence, the delicate balance between the positive net charge on protein surfaces and protein stability at pH 5.5 ensures the stability of bsAb A.

### Optimal stability and reversibility of self-associated aggregates for bsAb A achieved under mildly acidic conditions (pH 5.5) with minimal ionic strength

Although the four different buffering reagents (50 mM NaOAc, 50 mM NaCi, 50 mM NaPi, and 50 mM His-HCl) at pH 5.5 show relatively small impacts on both bsAb A stability and reversible self-association (Fig. 4), the His-HCl buffer was chosen for further investigation into the impact of ionic strength on bsAb A stability. This choice is due to the widespread use of histidine-HCl in formulating antibody drugs (Baek et al. 2019; Saurabh et al. 2022). The preserved stability conferred by histidine likely arises from its ability to provide both π-cation and hydrophobic interactions to proteins (Saurabh et al. 2022). Thus, instead of relying solely on intermolecular interactions among bsAb molecules themselves, histidine may offer alternative sources of interactions to proteins, potentially minimizing the likelihood of protein aggregation formation.

While the stability of bsAb A is reasonably well-maintained in 50 mM histidine-HCl at pH 5.5 across a broad range of salt concentrations (0–500 mM NaCl), its stability and reversibility of self-associated species are optimal in the absence of salt. This is likely because salts can shield the positively charged proteins, thereby diminishing repulsive intermolecular interactions. This reduction in repulsion could promote non-specific intermolecular interactions, shifting the equilibrium toward aggregate formation (Esfandiary et al. 2015), thereby suppressing protein stability and causing monomer loss. This rationale also explains the relatively maintained monomer concentrations observed in McIlvaine pH 5.5 during the initial 7-day incubation, whereas an increase in monomer concentrations was observed in four other buffers (Fig. 4). This evidence suggests that ionic strength, rather than buffer chemistry, may play a more significant role in the reversal of self-associated aggregates to monomers. This is supported by the fact that McIlvaine buffer at pH 5.5 also contains sodium phosphate (114 mM) and citric acid (43 mM), albeit at higher concentrations.

Our findings may appear to diverge from some previous studies, which suggest that salts typically exert a stabilizing effect for long-term stability at low ionic strength due to non-specific electrostatic interactions (Schermeyer et al. 2017). Our scenario is likely attributed to the increased hydrophobicity of bsAb A, stemming from the presence of the hydrophobic single-chain variable fragment (scFv) domain, in contrast to typical monoclonal antibodies. Biomolecules possessing elevated hydrophobic surfaces may exhibit preferential ion exclusion, thereby augmenting protein surface energy and ultimately facilitating aggregation (Arosio et al. 2012). It is essential to note, however, that ionic strength alone does not exclusively contribute to protein stability. Instead, it is intricately dependent on various factors such as pH, types of salts, protein concentrations, protein charge distribution, and amino acid sequences (Schermeyer et al. 2017; Arosio et al. 2012). These factors often interplay, which further complicating our understanding. For instance, studies have shown that three basic monoclonal antibodies (pI = 7.5–10) exhibit greater dissociation at high ionic strength near neutral pH (pH 6) than at low pH (pH 4.4), while lysozyme demonstrates repulsive self-interactions at low ionic strength across a wide range of pH values (pH 3–9) (Sule et al. 2012). Another study highlighted that aggregation propensity depends on both types of cations and anions at a pH of 4.0, whereas the types of cations do not play a role when the pH is reduced to 3.0 (Arosio et al. 2012). Therefore, fully rationalizing how ionic strength affects protein stability is often challenging and involves complexities.

### BsAb A stability is compromised at a higher protein concentration

Our findings indicate that the stability of bsAb A is compromised at higher protein concentrations (10 mg/mL) compared to lower concentrations (1 mg/mL). This is evident during the initial 5-day incubation period, where we observed a slight increase in %monomer, accompanied by a decrease in %HMW and monomer loss (Fig. 6). These results suggest a transition of portions of bsAb A in solution to irreversible aggregates, potentially making them undetectable by SEC-MALS.

The reduction in protein stability at higher concentrations is attributed to the promotion of protein–protein interactions (PPI). At elevated protein concentrations, molecules are in closer proximity, increasing the likelihood of molecular collisions and subsequent aggregation. A higher proportion of monomers tend to transition into partially unfolded structures, ultimately leading to the formation of fully denatured aggregates (Lee et al. 2020) (Fig. 8). This circumstance renders the self-associated species irreversible, shifting the equilibrium towards aggregate formation (Clarkson et al. 2016). This also rationalizes the reversal of bsAb A self-associated aggregates was not observed at the high protein concentration of 10 mg/mL but was detectable at the low protein concentration of 1 mg/mL (Fig. 6C).

Ultrahigh protein concentrations promote the formation of irreversible aggregates, posing challenges in maintaining protein stability during manufacturing, storage, and administration. This presents a significant hurdle in formulating biotherapeutics, especially those intended for subcutaneous administration, where medications often require exceedingly high protein concentrations, reaching several hundred mg/mL (Jiskoot et al. 2022). Such increased concentration results in elevated viscosity (Liu et al. 2005), leading to discomfort during injection (Berteau et al. 2015). To address this issue, specific excipients such as sugars (Sudrik et al. 2019; Svilenov and Winter 2020) and/or amino acids (Stolzke and Brandenbusch 2022) become essential components in the formulation buffers, alongside optimal pH and salt types/concentrations. These excipients play crucial roles in maintaining protein stability while reducing viscosity, thereby minimizing severe pain upon injection (Jiskoot et al. 2022; Berteau et al. 2015).

### Resilience of bsAb A stability under elevated temperature (40 °C)

Elevated temperatures typically promote protein aggregation by increasing molecular motion and collision frequency, thereby facilitating protein unfolding and nucleation (Fig. 8) (Wood et al. 2020). However, bsAb A demonstrates sustained stability at 40 °C, accompanied by monomer concentration increase over time, indicating its resilience to high temperatures. Moreover, the reversal of self-associated aggregates to monomers occurs at a higher rate when bsAb A is incubated at 40 °C compared to room temperature, obviously during the initial 3-day incubation (Fig. 7C).

The resilience of bsAb A at 40 °C helps to limit its aggregation events. Furthermore, higher temperatures supply the energy needed to disrupt non-specific interactions among self-associated species, thereby shifting the equilibrium towards the reversion of stable oligomers to monomers. It is noteworthy that bsAb A stability was assessed at a low protein concentration of 1 mg/mL. If higher protein concentrations were explored, it is likely that more prominent aggregates might be observed over time.

### Exploring implications for enhancing manufacturability of bsAb A

Our research identified favourable conditions for bsAb A that should be considered during process development to potentially enhance future manufacturing productivity. While bsAb A stability diminishes at pH 3.5 over an extended period (7 days), its purity remains largely intact within the initial 1-day incubation (Fig. 2A), with minimal formation of broadening monomeric SEC-MALS peaks (Fig. 3A) and minimal monomer loss (Fig. 2C). This observation supports stability of bsAb A during the purification process, particularly regarding Protein A chromatography elution and low pH hold for viral inactivation. Although it is beyond the scope of our study, exploring the impact of ionic strength on its stability at low pH to determine the threshold of salt concentration for the process is warranted.

Previous studies reveal that basic monoclonal antibodies maintain stability for up to 24 h under low pH conditions (pH 3–pH 4) in the absence of salt. Conversely, escalating NaCl concentrations (up to 500 mM) correlate with increased antibody instability, as evidenced by a rise in the average hydrodynamic radius (R_h_) over time (Arosio et al. 2011). Additionally, other research work suggests that high salt and low pH conditions may accelerate aggregate formation for selected antibodies by masking charge repulsion among protein molecules (Hari et al. 2010). Additionally, monomer recovery from stable oligomers occurs upon dilution of the protein from a high salt solution into a salt-free medium at the same low pH, attributed to heightened intramolecular charge repulsion (Skamris et al. 2016). These examples suggest that minimal salt concentration may generally support protein stability at low pH, as explained by minimal masking of repulsive electrostatic interactions among protein molecules. However, amino acid sequences and intrinsic properties of proteins can come to play, hence different stability profiles could be expected for different proteins.

Furthermore, our study indicates that bsAb A (at a total protein concentration of 1 mg/mL) remains stable under slightly acidic pH conditions (e.g., pH 5.5) with NaCl concentrations ranging from 0 to 500 mM over a 1-month period. Interestingly, the absence of salt provides the optimal condition for bsAb A stability. These findings are crucial for purification development and optimization, as well as formulation screening, aiming to maintain or even enhance bsAb A stability.

The favourable conditions identified in our study offer valuable insights, laying the groundwork for process and development considerations. However, it is essential to note that other stress conditions during manufacturing processes, such as shear forces during culturing and buffer exchanging processes, dynamic interactions with purification resins, and higher protein concentrations, may further compromise bsAb A stability. Therefore, further comprehensive evaluation and optimization are warranted during process development and formulation screenings.

## Conclusion

Our study unveils environmental conditions conducive to the remarkable stability of asymmetric bsAb A, housed within the Fab-scFv format. Maintaining a mildly acidic solution with minimal ionic strength proves crucial for preserving bsAb A stability, even at elevated temperatures of 40 °C while extreme pH conditions (pH 3.5 and pH 7.5) compromise the stability, likely through distinct molecular mechanisms. At pH 3.5, initial electrostatic repulsion aids reverting self-associated aggregates to monomers, but prolonged exposure leads to aggregation potentially via structural fluctuation and/or partial unfolding, while hydrophobic forces likely drive aggregation formation at pH 7.5. Purity traces, together with increase in monomer concentration profiles align with general molecular events for antibody aggregation formation, as well as the reversal of self-associated aggregates, being influenced by the environmental factors.

In terms of manufacturability, this study sheds light on potential buffer conditions for purification development, and formulation screenings. Despite the resilience observed in bsAb A stability, challenges persist at high protein concentrations, warranting further investigation. Future perspectives should encompass additional stability and formulation screenings at higher protein concentrations, along with the assessment of stability-indicating parameters (SIPs) such as short-term aggregation, thermal stability (T_onset_), and colloidal stability (diffusion interaction parameter, kD) to reflect actual manufacturability (Kenrick et al. 2014). These insights contribute significantly to biopharmaceutical manufacturing advancements.

### Supplementary Information

Supplementary Material 1.

## Data Availability

All data generated or analysed during this study are included in this published article and its supplementary information files.

## Appendix

1 $$ Relative\;monomer\;concentration\; \left( {RMC} \right) = \frac{{monomer\;concentration\;\left( {time = x} \right)}}{{monomer\; concentration\;\left( {time = 0\,{\text{h}}} \right)}}. $$

## Abbreviations

BiTE: Bispecific T-cell engager
bsAb: Bispecific antibody
HC: Heavy chain
LC: Light chain
HCCF: Harvest cell culture fluid
His-HCl: Histidine-HCl
NaCi: Sodium citrate
NaOAc: Sodium acetate
NaPi: Sodium phosphate
PPI: Protein–protein interactions
Rh: Hydrodynamic radius
RMC: Relative monomer concentration
RSA: Reversible self-association
scFv: Single chain variable fragment
