# Supplementary material for: Investigation on environmental factors contributing to bispecific antibody stability and the reversal of self-associated aggregates
Source: Bioresour Bioprocess. 2024 Aug 23;11(1):82. doi: 10.1186/s40643-024-00796-y (PMC11343937; doi:10.1186/s40643-024-00796-y)
Supplement: Supplementary file 1 — Supplementary Material 1. [file 40643_2024_796_MOESM1_ESM.docx]

**Supplementary material**

Investigation on Environmental Factors Contributing to Bispecific Antibody Stability and the Reversal of Self-Associated Aggregates

Nattha Ingavat^1^, Nuruljannah Dzulkiflie^1^, Jia Min Liew^1^, Xinhui Wang^1^, Eunice Leong^2^, Han Ping Loh^3^, Say Kong Ng^2^, Yuansheng Yang^3^, Wei Zhang^1^*

^1^Downstream Processing Group, Bioprocessing Technology Institute, Agency for Science, Technology and Research (A*STAR), Singapore

^2^Animal Cell Bioprocessing Group, Bioprocessing Technology Institute, Agency for Science, Technology and Research (A*STAR), Singapore

^3^Cell Line Development Group, Bioprocessing Technology Institute, Agency for Science, Technology and Research (A*STAR), Singapore

*Correspondence:

Wei Zhang, [zhang_wei@bti.a-star.edu.sg](mailto:zhang_wei@bti.a-star.edu.sg)


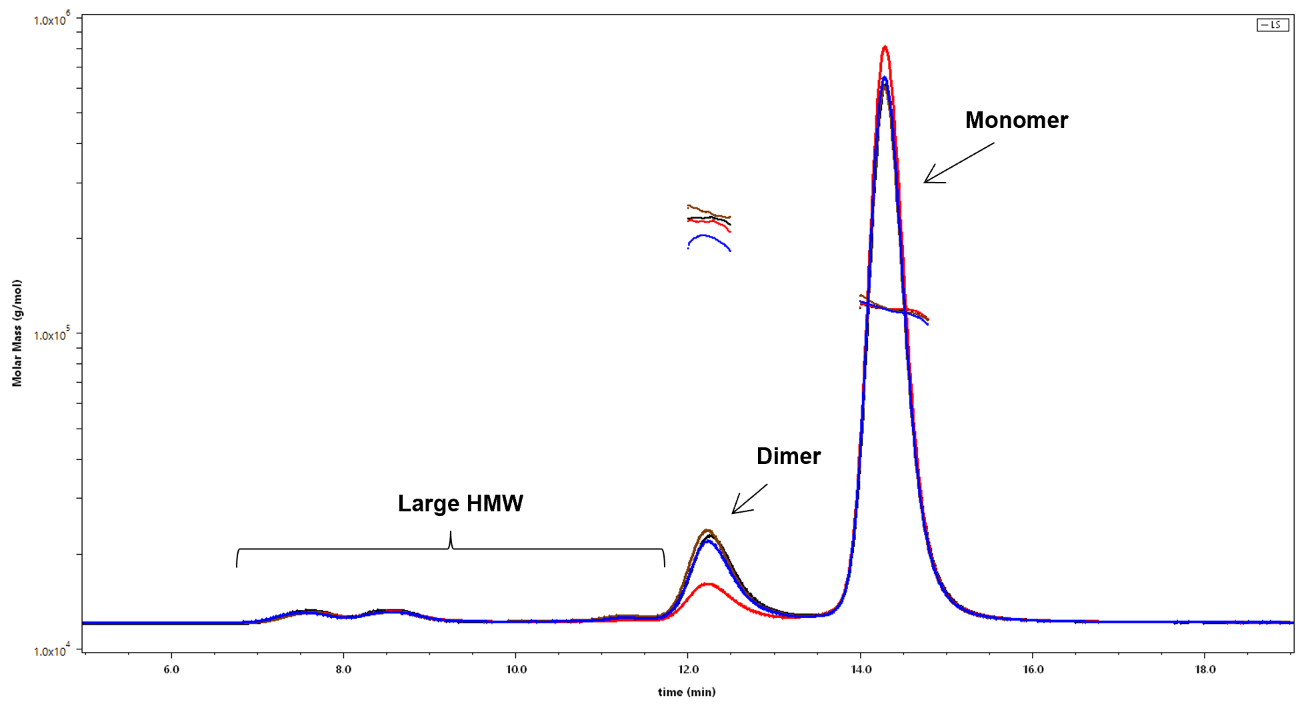


Supplementary Figure 1. Overlays of SEC-MALS profiles of bsAb A post-ProA pH 6.5 (black); post-ProA buffer-exchanged into buffer pH 3.5 (red), pH 5.5 (blue), and pH 7.5 (brown) (time = 0 h).


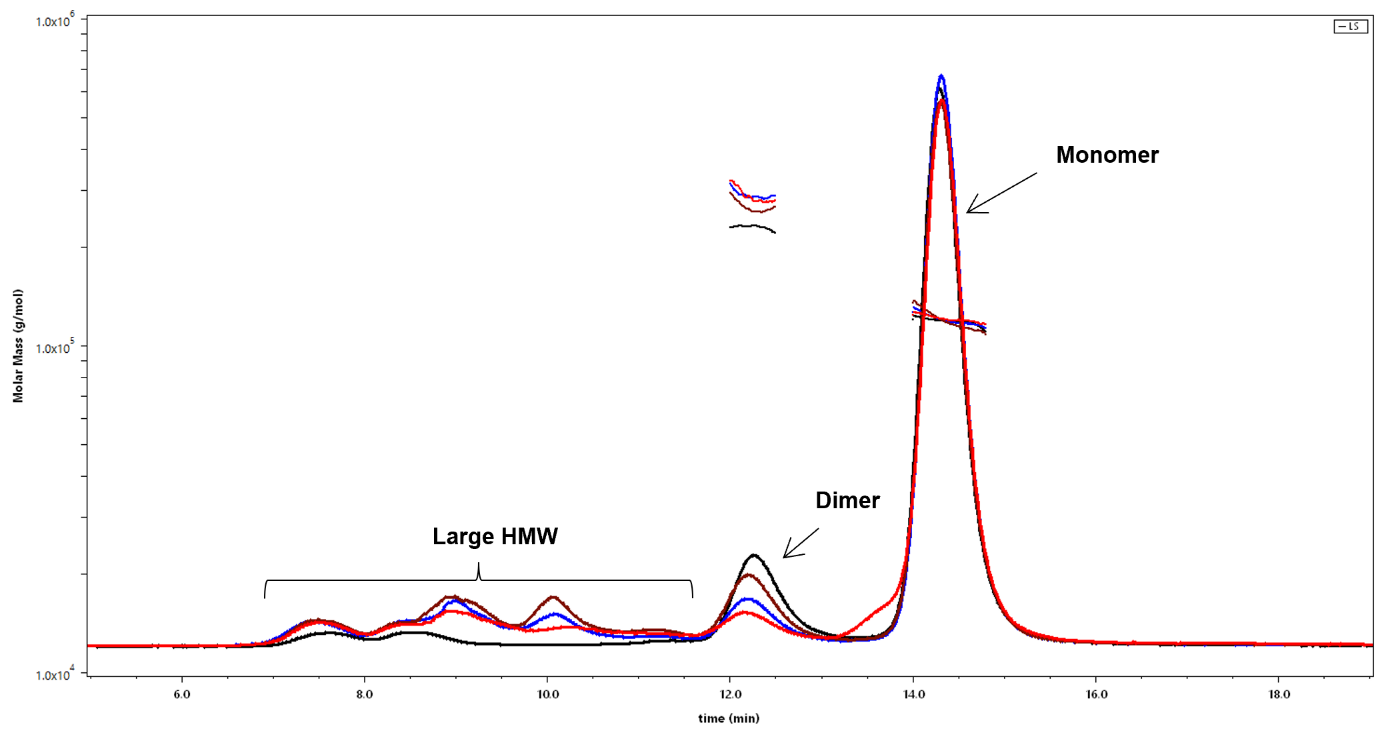


Supplementary Figure 2. Overlays of SEC-MALS profiles of bsAb A post-ProA pH 6.5 (black); post-ProA buffer-exchanged into buffer pH 3.5 (red), pH 5.5 (blue), and pH 7.5 (brown) after 7-day incubation (Day 7).
